# Applicability of Quality Indicators for Appropriate Antibiotic use in Outpatient Parenteral Antimicrobial Therapy (OPAT): A Point Prevalence Survey

**DOI:** 10.3389/fphar.2021.713882

**Published:** 2021-08-25

**Authors:** Pablo March-López, Inés Arancibia Freixa, Mireia Martinez Gil, Gastón Araujo Espinoza, Lidia Ortega Polonio, Elisabeth Cecilia Paredes, Montserrat Carrasco Sanchez, Cristina Sangrador, Júlia Pardo, Jordi Nicolás, Esther Calbo

**Affiliations:** ^1^ Hospital Pharmacy, Pharmacy Department, Hospital Universitari Mútua Terrassa, Barcelona, Spain; ^2^ School of Medicine, Universitat Internacional de Catalunya, Barcelona, Spain; ^3^ Hospital at Home Unit Physician, Mútua Terrassa, Barcelona, Spain; ^4^ Hospital at Home Unit Nurse, Hospital Universitari Mútua Terrassa, Barcelona, Spain; ^5^ Pharmacy Department, Universitat de Barcelona, Barcelona, Spain; ^6^ Infectious Diseases Unit, Hospital Universitari Mútua Terrassa, Barcelona, Spain

**Keywords:** opat, hospital at home, antimicrobial resist ance, antibiotics, quality indicator

## Abstract

The ability to measure the quality of antibiotic prescription is a critical element in any antimicrobial stewardship programme. The aim of this study was to evaluate the clinimetric properties of 33 quality indicators (QIs) developed to assess Outpatient Parenteral Antimicrobial Therapy (OPAT) and to identify potential room for improvement in a hospital-at-home (HaH) unit. Study performed in a healthcare district in Barcelona, Spain with 260,657 inhabitants, nine primary healthcare centres, a 400-bed acute care teaching hospital, and an HaH unit. We studied 33 QIs on appropriate antibiotic use and classified them as qualitative or quantitative. Quantitative QIs were further categorized as measurable or non-measurable depending on the availability of data in the patients’ medical records. Data from 202 OPAT episodes in 192 patients were assessed. Adherence was found for 22 of the 24 qualitative QIs analyzed; the other two showed room for improvement. Four of the nine quantitative indicators were non-measurable. High adherence rates were achieved for QI-17 “The OPAT plan should be documented” (84.65%), QI-26 “The OPAT treatment plan should include choice, dose, frequency, duration and follow-up plan” (79.70%), and QI-33 “The team should document clinical response” (94.55%). Adverse events were documented in just 1.98% of cases (QI-32) and 92.57% patients were classified as alive on discharge (QI-24). The QIs evaluated were applicable to clinical practice and proved useful for identifying areas with room for improvement in our setting and for guiding the design of future interventions with specific objectives.

## Introduction

Outpatient parenteral antimicrobial therapy (OPAT) has been defined as the provision of parenteral antimicrobial therapy in at least two doses on different days without intervening hospitalization (Tice et al., 2004). It allows patients who would typically need to be hospitalized to receive parenteral antimicrobials at home, thereby increasing the availability of hospital beds by shortening or even eliminating hospital stays (Barr et al., 2012).

Hospital-at-home (HaH) programmes originated in the United States in the mid-1970s, partly in response to gaps in medical insurance coverage that made the cost of inpatient care prohibitive for certain patients requiring intravenous antibiotic therapy (Williams et al., 2015). In Spain, the first HaH unit was created by Hospital Gregorio Marañón in Madrid in 1981. The foundation of the Spanish Society for Home Hospitalization (SEHAD) in 2006 represented an important step forward in the development of HaH programmes across the country. As of January 2021, there were 105 active HaH units in Spain (Sociedad Española de HospitalizaciónDomiciliaria, 2020).

The current COVID-19 pandemic is one of the greatest public health crises of our time (European Centre for Disease Prevention and Control, 2019; World Health Organization, 2020a), and has forced health systems worldwide to urgently find ways to relieve pressure on overstretched hospitals and increase bed availability. One outcome has been an increase in the use of HaH programmes, which have proven to be both an efficient and safe option for providing care to selected at-risk patients (Coloma and Nicolás, 2020; Moghadas et al., 2020; Pericàs et al., 2020; Shoukat et al., 2020). OPAT patients, for example, are a COVID-19 risk group because of their susceptibility to severe infections and extensive contact with the healthcare system (Mansour et al., 2020). Regardless of how suitable a patient may seem for inclusion in an OPAT program, there is always the risk of important steps being forgotten or misapplied. Choosing a regimen that is suited to the capabilities of each patient and ensuring correct education and instruction are crucial to the success of any program (Halilovic et al., 2014).

Increasing the number of patients assigned to OPAT care also provides an opportunity to improve antimicrobial stewardship (AS). OPAT-specific quality indicators (QIs), however, are needed, as the ability to measure the quality of antibiotic prescriptions is a critical element in any AS programme. There are three main types of QIs: 1) structure indicators, to assess the organization of the healthcare setting; 2) process indicators, to assess the care received and delivered; and 3) outcome indicators, to assess the consequences of a given intervention (Donabedian, 1988).

In 2019, Berrevoets et al. (Berrevoets et al., 2020) and a multidisciplinary team of international experts developed a set of 33 generic QIs for appropriate OPAT use in adults. The QIs were classified into four categories: organization, initiation, continuation, and outcome. The experts, however, recognized that these QIs first needed to be tested for applicability in different clinical practice settings. One way of doing this is to test their clinimetric properties.

The aim of this study was to assess the usefulness of the QIs developed by Berrevoets et al. (Berrevoets et al., 2020) for identifying room for improvement in OPAT use and guiding the design of strategies for enhancing AS within an HaT OPAT programme in a health district of a high-income country with a public healthcare system (Spain).

## Materials and Methods

This was an observational study performed in a health district with nine primary care centers, a 400-bed acute care teaching hospital, and an HaH unit that provides active home care to patients who would otherwise need acute medical care in a hospital setting. The OPAT programme is one of the unit’s main activities. It serves a population of 260,000 inhabitants and is run by three physicians, three nurses, and a clinical pharmacist. The Spanish national healthcare system offers universal coverage to a population of 46.4 million inhabitants (2016).

### QIs and Definitions

The 33 QIs on appropriate antibiotic use developed by Berrevoets et al. (Berrevoets et al., 2020) were classified as qualitative or quantitative. Indicators that could be rated as adhered to (YES) or not adhered to (NO) were classified as qualitative, while those for which the degree of adherence could be measured were classified as quantitative. Quantitative indicators were further classified as measurable or non-measurable depending on whether or not the patients medical records contained the information necessary for assessment. Of the 33 QIs evaluated, 24 were qualitative (1–16, 18–21, 27–30) and nine were quantitative (17, 22–26, 32, 33). Four of the quantitative indicators were non-measurable (22, 23, 25, 31).

For QI-17 “The OPAT plan should be documented in the discharge summary”, the patients medical records were checked to see if the discharge summary contained information on antibiotic choice, dose, frequency of administration, and duration of OPAT. Five survival status at discharge categories were analyzed for QI-24 “The survival status of patients who received OPAT should be documented”: patient alive, died of infection, died of other causes, lost to follow-up, and status unknown. The patients’ medical records were also reviewed to check adherence to QI-26 “The OPAT treatment plan should include the following items: choice, dose, frequency, duration, and follow-up plan”, QI-32 “The OPAT team should document adverse events related to devices, antibiotic use, and toxicity”, and QI-33 “The OPAT team should document clinical response to antimicrobial management”.

### Study Population and Data Collection

All patients who participated in the HaH OPAT programme between July 2019 and June 2020 were included. Patients treated with an antibiotic for fewer than 2 days were excluded following the Infectious Diseases Society of America definition of OPAT (Tice et al., 2004). To characterize the demographics of the programme participants, we collected information on type of infection, duration of OPAT within HaH program, choice of antibiotics, and microorganisms isolated.

The data required to assess the clinimetric properties of the QIs were extracted from the patients electronic medical records. Clinical and laboratory data were extracted from medical and nursing records and medication charts. Antibiotic prescriptions were identified by ATC codes and infections by International Classification of Disease codes (ninth version) (MSCBS, 2014; World Health Organization, 2020b). All data were collected and processed by members of the HaH team.

Adherence to individual QIs was defined as the percentage of patients for whom the indicator was met. Potential room for improvement measures the sensitivity of a potential indicator to identify areas in which antibiotic prescription can be improved. For quantitative indicators, it was calculated as 100% minus the adherence rate in all cases except *QI-24* “The survival status of patients receiving OPAT should be documented” and QI-32 “The OPAT team should document adverse events related to devices, antibiotic use, and toxicity”, where these calculations are impossible.

The patients in our cohort study all received OPAT initiated within the hospital but delivered at home, which is in line with the definition provided by Chapman et al. in the United Kingdom consensus statement on good practice recommendations for OPAT in adults (Chapman et al., 2012) and applied by Berrevoets et al. (Berrevoets et al., 2020): “a method for delivering intravenous antimicrobials in the community or outpatient setting, as an alternative to inpatient care”.

### Statistical Analysis

Proportions, interquartile range, and standard deviations were used for descriptive statistics. Analyses were performed using Stata 13 (Stata Corporation, College Station, TX, United States).

## Results

Between July 2019 and June 2020, the HaH unit provided care to 717 patients for 821 episodes. Over a quarter of these patients (n = 192, 26.78%) received OPAT care (202 episodes); 66.83% of the patients were men and the mean overall age was 66.65 years (SD 16.65). The mean duration of the OPAT plan was 9.39 days (interquartile range, 4–11 days).

The most common diagnoses were genitourinary and respiratory tract infections (Table 1). The most prevalent microorganisms were Gram-negative bacteria (44.06%), including *Escherichia coli* harboring extended spectrum beta-lactamase (15.94%) (Table 2). Ceftriaxone (39.11%) and carbapenems (34.50%) were the main antibiotics administered (Table 3).

**TABLE 1:** Infections treated with outpatient parenteral antimicrobial therapy.

Type of infection	N	**%** ^3^
Genitourinary	101	50.3
Urinary tract infection^1^	77	38.3
Pyelonephritis^2^	11	5.5
Prostatitis	11	5.5
Epididymo-orchitis	2	1.0
Respiratory	47	23.4
Pneumonia	21	10.5
Acute bronchitis	10	5.0
Exacerbated COPD	7	3.5
Acute bronchitis	4	2.0
Bronchiectasis	3	1.5
Respiratory tract infection	1	0.5
Massive bronchial aspiration	1	0.5
Skin and soft tissue	22	10.9
Surgical wound infection	11	5.5
Wound infection	4	2.0
Abscess	2	1.0
Cellulitis	2	1.0
Pressure ulcer infection	2	1.0
Chondritis	1	0.5
Abdominopelvic	11	5.5
Diverticulitis	3	1.5
Cholangitis	2	1.0
Peritonitis	2	1.0
Pancreatic infection	1	0.5
Intrahepatic infection	1	0.5
Postsurgical intra-abdominal collection	1	0.5
Acute cholecystitis	1	0.5
Intravascular infections	10	5.0
Bloodstream infections^1^ ^,^ ^2^	10	5.0
Cardiac	5	2.5
Endocarditis	4	2.0
Aortic stent infection	1	0.5
Musculoskeletal	2	1.0
Osteomyelitis	1	0.5
Prosthetic joint infection	1	0.5
Other infection	3	1.5
Sepsis	2	1.0
Febrile neutropenia	1	0.5

1 ^,2^Patients with two infections.

3 %of all diagnoses (n = 201).

**TABLE 2:** Microorganisms isolated.

Microorganism isolated	N	**%**
Gram-negative bacteria	93	44.1
*Escherichia coli* harboring ESBL^1^	32	15.8
*Pseudomonas aeruginosa*	0	9.9
*Escherichia coli* ^2^	20	9.9
*Klebsiella pneumoniae* harboring ESBL^1^	8	4.0
*Klebsiella pneumoniae*	4	2.0
*Pseudomonas aeruginosa*, multidrug-resistant	3	1.5
*Proteus* mirabillis	2	1.0
*Pseudomonas* putida	1	0.5
*Serratia* mercescens	1	0.5
*Citrobacter freundii*	1	0.5
*Acinetobacter* baumannii^2^	1	0.5
Gram-Positive	29	13.4
*Enterobacter cloacae*	6	3.0
*Enterococcus faecalis* ^3^ ^,^ ^2^	6	3.0
*Streptococcus pneumoniae*	4	2.0
*Staphylococcus*epidermidis	3	1.5
*Staphylococcus aureus*	3	1.5
Strepptococcus gallolyticus ssp.pasteurianus	2	1.0
Methicillin-resistant *Staphylococcus aureus*	1	0.5
Strepptococcus lutetiensis	1	0.5
*Staphylococcus* hominis	1	0.5
Strepptococcus agalactiae	1	0.5
*Enterococcus* faecium	1	0.5
Others		
Negative culture	56	27.7
No culture	23	11.4
Contaminated culture	1	0.5
Total samples	199	100
Total	202^3^	100

ESBL: extended spectrum beta-lactamase.

2 Both microorganisms isolated in the same sample.

3 %of total (202).

**TABLE 3:** Outpatient parenteral antimicrobial antibiotics.

Antibiotic	N	**%** ^**2**^
Combinations of penicillins, incl. beta-lactamase inhibitors (J01CR)		
Piperacillin/tazobactam	6	3.0
Amoxicillin/clavulanic acid	5	2.5
Beta-lactamase resistant penicillins (J01CF)		
Cloxacillin	1	0.5
First-generation cephalosporins (J01DB)		
Cefazolin	2	1.0
Third-generation cephalosporins (J01DD)		
Ceftazidime	8	4.0
Ceftriaxone	77	38.5
Fourth-generation cephalosporins (J01DE)		
Cefepime	3	1.5
Carbapenems (J01DH)		
Ertapenem^1^	61	30.2
Meropenem	7	3.5
Imipenem/cilastatin	1	0.5
Other aminoglycosides (J01GB)		
Amikacin	20	9.9
Fluoroquinolones (J01MA)		
Levofloxacin	2	1.0
Glycopeptide antibacterials (J01XA)		
Teicoplanin	1	0.5
Vancomycin	1	0.5
Other antibacterials (J01XX)		
Daptomycin^1^	5	2.5
Total treatments	199	100
Total antibiotics	200	100

1 One patient on dual therapy.

2 % of total number of antibiotics (n = 200).

Twenty-two of the 24 qualitative QIs (1–12, 14–16, 18–21, 27, 28, 30) were adhered to and two (13, 29) showed room for improvement.

In the group of quantitative QIs, there were five measurable indicators (17, 24, 26, 32, 33) and four non-measurable indicators (22, 23, 25, 31). High adherence was observed for QI-17 “The OPAT plan should be documented in the discharge summary” (adherence rate, 84.65%), QI-26 “The OPAT treatment plan should include the following items: choice, dose, frequency, duration and follow-up plan” (79.70%), and QI-33 “The OPAT team should document clinical response to antimicrobial management” (94.55%).

Survival status at discharge was documented for all patients (QI-24); 92.57% were classified as patient alive, 4.95% as readmitted to hospital, 1.49% as died of other causes, and 0.99% as died of infection. None of the patients were classified as lost to follow-up or status unknown*.* Adverse events were documented for just 1.98% of patients (QI-32).

The 33 QIs, together with their classification and adherence ratings, are listed in Table 4.

**TABLE 4:** Quality Indicators: Classification, Adherence, and Room for Improvement.

	Quality Indicator	Classification	Adherence	Room for improvement
1	There should be a structured OPAT program to provide a framework for safe and effective care.	Qualitative	Yes	N/A
2	The OPAT program should be part of an antimicrobial stewardship program.	Qualitative	Yes	N/A
3	There should be a formal OPAT care team.	Qualitative	Yes	N/A
	The OPAT care team should include: an infectious diseases specialist or physician knowledgeable about infectious diseases and the use of antimicrobials in OPAT, nurse expert in intravenous therapy, access devices and OPAT, pharmacist knowledgeable about OPAT.			
4	The OPAT team should have an identifiable medically qualified lead clinician who has identified time for OPAT in their job plan.	Qualitative	Yes	N/A
5	There should be a guideline for vascular access systems used, including site care.	Qualitative	Yes	N/A
6	There should be a policy on patient selection criteria for OPAT.	Qualitative	Yes	N/A
	The following key aspects of patient selection should be taken into account: patients are willing to comply with follow-up plan, there is appropriate home environment/adequate support, there are no clinical contraindications to discharge from hospital, no iv-oral switch possible, patient and caregiver understanding.			
7	There should be a policy outlining responsibilities of OPAT team members.	Qualitative	Yes	N/A
8	The OPAT ID physician should specify infection-related inclusion and exclusion criteria for OPAT.	Qualitative	Yes	N/A
9	A competent member of the OPAT team should perform the initial assessment.	Qualitative	Yes	N/A
10	OPAT ID physician consultation should take place prior to intravenous access device placement.	Qualitative	Yes	N/A
11	Patients and caregiver should be given the opportunity to decline or accept this mode [OPAT] of therapy.	Qualitative	Yes	N/A
12	Patients and family should be informed about OPAT.	Qualitative	Yes	N/A
	The information they get should at least include: benefits, side-effects, potential complications, vascular access/sterile techniques, responsible physician until patients seen in clinic, instructions for emergencies, antimicrobial use, patient responsibilities, nature of OPAT, contact lists, use of antibiotics (e.g. storage conditions).			
13	In case of self-administration, both the OPAT nurse specialist and patient/caregiver must be satisfied of the patient’s/ caregiver competence and this should be documented.	Qualitative	No	Yes
14	There should be a mechanism in place for urgent discussion and review of emergent clinical problems during OPAT according to clinical need.	Qualitative	Yes	N/A
15	There should be a system in place for rapid communication between the patient and team members.	Qualitative	Yes	N/A
16	There should be communication between the OPAT team and other stakeholders involved.	Qualitative	Yes	N/A
	These stakeholders should at least include: general practitioner, community team (when appropriate), referring clinician. At a minimum communication with stakeholders should include notification of acceptance into the OPAT program, notification of completion of therapy and notification of complications.			
17	The OPAT plan should be documented in the discharge summary.	Quantitative/measurable	84.6%	15.35%
18	The OPAT treatment plan is the responsibility of the OPAT ID physician, following discussion with the referring clinician.	Qualitative	Yes	N/A
19	Laboratory results should be delivered to physicians within 24 h after obtaining material for testing.	Qualitative	Yes	N/A
20	The treatment plan of patients receiving in excess of 1 week of antimicrobial therapy should be regularly reviewed by the OPAT specialist nurse and physician (narrow spectrum antibiotics, iv-oral switch) in conjunction/consultation with the referring specialist as necessary.	Qualitative	Yes	N/A
21	The intravascular access device should be removed after end of therapy, if not needed for another reason.	Qualitative	Yes	N/A
22	The program outcome of patients receiving OPAT should be monitored (e.g. therapy completed as planned/therapy not completed as planned because of … ).	Quantitative/non-measurable	N/A	Yes
23	Antibiotic use of patients receiving OPAT should be monitored (e.g. completed as planned/not completed as planned because of … ).	Quantitative/non-measurable	N/A	Yes
24	The survival status of patients receiving OPAT should be documented (e.g. patient alive, died of infection, died of other causes, lost to follow-up, or status unknown).	Quantitative/ measurable	Patient alive: 92.6%	N/A
			Hospital readmission: 5.0%	
			Died of other causes: 1.5%	
			Died of Infection: 1.0%	
			Lost to follow-up: 0%	
			Status unknown: 0%	
25	The satisfaction status/experiences of patients receiving OPAT should be monitored.	Quantitative/non-measurable	N/A	Yes
26	The OPAT treatment plan should include the following items: choice, dose, frequency, duration and follow-up plan.	Quantitative/ measurable	79.7%	20.3%
27	The OPAT team should select the drug delivery device in agreement with the home health agency.	Qualitative	Yes	N/A
28	In case of self-administration, patients or caregivers should be trained in the administration of iv antibiotics.	Qualitative	Yes	N/A
29	The OPAT team should monitor quality indicators for OPAT care and make these data available.	Qualitative	No	Yes
30	Patient educational material should be available in written form or in multimedia documents.	Qualitative	Yes	N/A
31	There should be an OPAT treatment and monitoring plan.	Quantitative/non- measurable	N/A	N/A
	The OPAT treatment and monitoring plan should at least include:			
	Indication, antibiotic name, dose, frequency, duration, type of administration (e.g. continuous infusion or bolus infusion), access device used (e.g. PICC, tunneled catheter).			
**32**	The OPAT team should document adverse events related to devices, antibiotic use and toxicity.	Quantitative/measurable	2.0%	N/A
**33**	The OPAT team should document clinical response to antimicrobial management.	Quantitative/measurable	94.6%	5.4%

N/A, not applicable.

## Discussion

We have evaluated adherence and room for improvement for a set of QIs in a cohort of OPAT patients treated in Spain, a high-income country with a national public health system. Our findings highlight the importance of testing the clinimetric properties of QIs before using them in routine practice. To our knowledge, this is the first study to assess the performance properties of the QIs developed by Berrevoets et al. for appropriate OPAT use in adults (Berrevoets et al., 2020).

The QIs developed by Berrevoets et al. (Berrevoets et al., 2020) are, to our knowledge, the first set of generic QIs to be designed for OPAT units. The authors, however, did not validate the QIs or perform any clinimetric evaluations. They acknowledged this limitation and proposed that their generic set of QIs be assessed and adapted to national specificities and guidelines as needed.

Our results show high adherence to the vast majority of QIs analyzed. Just two of the qualitative indicators showed room for improvement: QI-29 “The OPAT team should monitor quality indicators for OPAT care and make these data available”, which is the purpose of this present study, and QI-13 “In case of self-administration, both the OPAT nurse specialist and patient/caregiver must be satisfied of the patient’s/ caregiver competence and this should be documented”. In this second case, based on our interviews with the OPAT team, we determined that patients receive the necessary training on self-administration but that there are no mechanisms in place for documenting satisfaction with patient/caregiver competence. The observation of low adherence to certain indicators highlights the need to improve data recording systems to detect areas with room for improvement. Inclusion of a purpose-designed form in patients’ clinical records could improve adherence.

High adherence was achieved for the five measurable quantitative QIs, indicating little room for improvement. Our analysis of QI-24 “The survival status of patients receiving OPAT should be documented” shows that most patients were discharged successfully and that mortality was very low, indicating the adequacy of the selection criteria for OPAT in our setting (stable patients with a low risk of mortality). In relation to QI-32 “The OPAT team should document adverse events (AE) related to devices, antibiotic use, and toxicity”, we detected a very low rate of adverse events (1.98%), supporting previous reports showing the effectiveness of HaH units for OPAT. It is impossible to determine the true rate of adverse events, but rates related to the use of antibiotics tend to lie between 6.0 and 18.0% (Keller et al., 2018; Sriskandarajah et al., 2018; Quintens et al., 2020). The low rate in our study is probably due to underreporting. The introduction of active pharmacovigilance to complement passive reporting would help capture a truer picture of adverse effects.

In a recent systematic review on the safety and effectiveness of OPAT, Sriskandarajah et al.^24^ reported a cure or treatment success rate of greater than 80% in more than 88% of the studies, with drug-related adverse events ranging from 0 to 30.2%. Considering that HaH activities are likely to continue to increase, the QIs that we have analyzed will be useful for evaluating both patient safety and OPAT outcomes. Future audits can be improved by including a specific section in the discharge summary covering information relating to the four non-measurable QIs in our series (22, 23, 25, and 31).

Our findings have added to the current evidence in several respects. To our knowledge, this is the first study to test the clinimetric properties of the set of standardized QIs developed by Berrevoets et al. using the Delphi process, conferring robustness and validity (Moghadas et al., 2020). We also believe that we are the first healthcare organization to report on the use of OPAT-specific QIs and that our findings are applicable to countries with a public health system and a similar economic situation to Spain. Finally, our results have highlighted the need for improved data recording systems and will help ensure the continued provision of high-quality OPAT at our hospital.

This study also has some potential limitations. First, the work involved in manually retrieving the data needed to assess the clinimetric properties of the QIs was a laborious, time-consuming task that would probably be unfeasible outside the scope of a research project. It may also have influenced the objectivity of our findings. Automated data systems would facilitate data collection and reduce the risk of errors and bias. Second, we were unable to measure certain QIs due to missing data in the discharge summary. Third, care should be taken when extrapolating our findings to other settings, as this was an observational study of a single HaH program run by three nurses and one clinical pharmacist at a single healthcare organization providing care to a population of 260,000 inhabitants. Finally, the study was performed during the COVID-19 pandemic. It would be interesting to perform a similar study following the pandemic to investigate its influence on the HaH OPAT programme at our hospital.

## Conclusion

We have tested the applicability and feasibility of QIs for appropriate antibiotic use in a real-life HaH OPAT program. Our analysis helped identify areas with room for improvement and in particular, will enable us to improve our electronic reporting systems and maintain a high-quality OPAT program.

## Data Availability

The original contributions presented in the study are included in the article/supplementary material; further inquiries can be directed to the corresponding authors.
